# Prediction of Hypertension in the Pediatric Population Using Machine Learning and Transfer Learning: A Multicentric Analysis of the SAYCARE Study

**DOI:** 10.3389/ijph.2025.1607944

**Published:** 2025-03-11

**Authors:** Keisyanne Araujo-Moura, Letícia Souza, Tiago Almeida de Oliveira, Mateus Silva Rocha, Augusto César Ferreira De Moraes, Alexandre Chiavegatto Filho

**Affiliations:** ^1^ Department of Epidemiology, School of Public Health, University of São Paulo, São Paulo, Brazil; ^2^ Department of Statistic, State University of Paraíba, Campina Grande, Paraíba, Brazil; ^3^ School of Public Health in Austin, Department of Epidemiology, Michael and Susan Dell Center for Healthy Living, Texas Physical Activity Research Collaborative (Texas PARC), University of Texas Health Science Center at Houston, Houston, TX, United States

**Keywords:** hypertension, machine learning, children, adolescents, public health

## Abstract

**Objective:**

To develop a machine learning (ML) model utilizing transfer learning (TL) techniques to predict hypertension in children and adolescents across South America.

**Methods:**

Data from two cohorts (children and adolescents) in seven South American cities were analyzed. A TL strategy was implemented by transferring knowledge from a CatBoost model trained on the children’s sample and adapting it to the adolescent sample. Model performance was evaluated using standard metrics.

**Results:**

Among children, the prevalence of normal blood pressure was 88.9% (301 participants), while 14.1% (50 participants) had elevated blood pressure (EBP). In the adolescent group, the prevalence of normal blood pressure was 92.5% (284 participants), with 7.5% (23 participants) presenting with EBP. Random Forest, XGBoost, and LightGBM achieved high accuracy (0.90) for children, with XGBoost and LightGBM demonstrating superior recall (0.50) and AUC-ROC (0.74). For adolescents, models without TL showed poor performance, with accuracy and recall values remaining low and AUC-ROC ranging from 0.46 to 0.56. After applying TL, model performance improved significantly, with CatBoost achieving an AUC-ROC of 0.82, accuracy of 1.0, and recall of 0.18.

**Conclusion:**

Soft drinks, filled cookies, and chips were key dietary predictors of elevated blood pressure, with higher intake in adolescents. Machine learning with transfer learning effectively identified these risks, emphasizing the need for early dietary interventions to prevent hypertension and support cardiovascular health in pediatric populations.

## Introduction

Hypertension (HTN) is a prevalent medical condition, affecting approximately one in four individuals worldwide, and represents a significant risk factor for heart disease, stroke, kidney failure, and mortality [1]. It is the leading global cause of morbidity and mortality associated with cardiovascular diseases (CVD). The complexity of HTN lies not only in its widespread prevalence but also in its asymptomatic progression during early stages, often delaying timely diagnosis and treatment [2].

The global burden of hypertension has increased significantly over recent decades, rising from 594 million cases in 1975 to 1.13 billion in 2015, with the majority of this growth occurring in low- and middle-income countries. This rise is primarily attributed to aging populations, lifestyle modifications, and demographic expansion. Approximately 13% of all deaths globally are associated with hypertension, underscoring its role as a major public health challenge that affects all sectors of society [3, 4].

Evidence from pathophysiological and epidemiological studies highlights the association between hypertension during childhood and an increased risk of hypertension and adverse cardiovascular events in adulthood. However, identifying HTN in pediatric populations poses unique challenges due to the dynamic changes in growth and development that complicate standardization of definitions and measurements, as well as the assessment of cardiovascular outcomes in children compared to adults [5, 6].

Data on the prevalence of elevated blood pressure in children are often derived from the National Health and Nutrition Examination Survey (NHANES) and are frequently limited to a single blood pressure measurement session [7, 8]. Since 1988, research has documented a rising prevalence of elevated blood pressure in children, including both hypertension and prehypertension, with rates consistently higher among boys (15%–19%) compared to girls (7%–12%). Preventive strategies targeting individuals and high-risk groups are essential to mitigate the long-term consequences of HTN. The necessity for early identification of at-risk individuals has spurred increasing interest in predictive models for hypertension risk [9, 10].

In recent years, artificial intelligence (AI) has emerged as a transformative tool in healthcare, demonstrating its utility in managing a variety of clinical conditions [11, 12]. AI facilitates the development of accurate risk prediction models for HTN by integrating traditional cardiovascular risk factors with multi-omic, socioeconomic, behavioral, and environmental data, thereby enabling the formulation of personalized treatment approaches [13].

A promising innovation within the domain of AI is transfer learning (TL), a machine learning (ML) technique that repurposes models trained for one task as a foundation for related tasks. TL is particularly advantageous in scenarios where the target dataset is limited, a common challenge in pediatric health research. For instance, TL has been successfully employed in studies predicting diabetes and cardiovascular diseases by leveraging large datasets to enhance predictive accuracy in smaller, specific populations [14]. Previous studies have demonstrated the efficacy of TL in predicting glucose levels among patients with type 1 diabetes, where it substantially improved model performance despite limited data availability. In the context of HTN, TL has the potential to transfer knowledge from models trained on extensive adult datasets to pediatric populations, thereby addressing the scarcity of data in children and adolescents [15, 16].

Given the increasing prevalence of elevated blood pressure in the pediatric population and its significant long-term health implications, this study aims to develop a machine learning model employing transfer learning to predict hypertension in children and adolescents in South America. By leveraging data from a comprehensive pediatric database, this study seeks to improve the accuracy of predictions and facilitate early interventions in these populations.

## Methods

### Study Design

**FIGURE 1:**
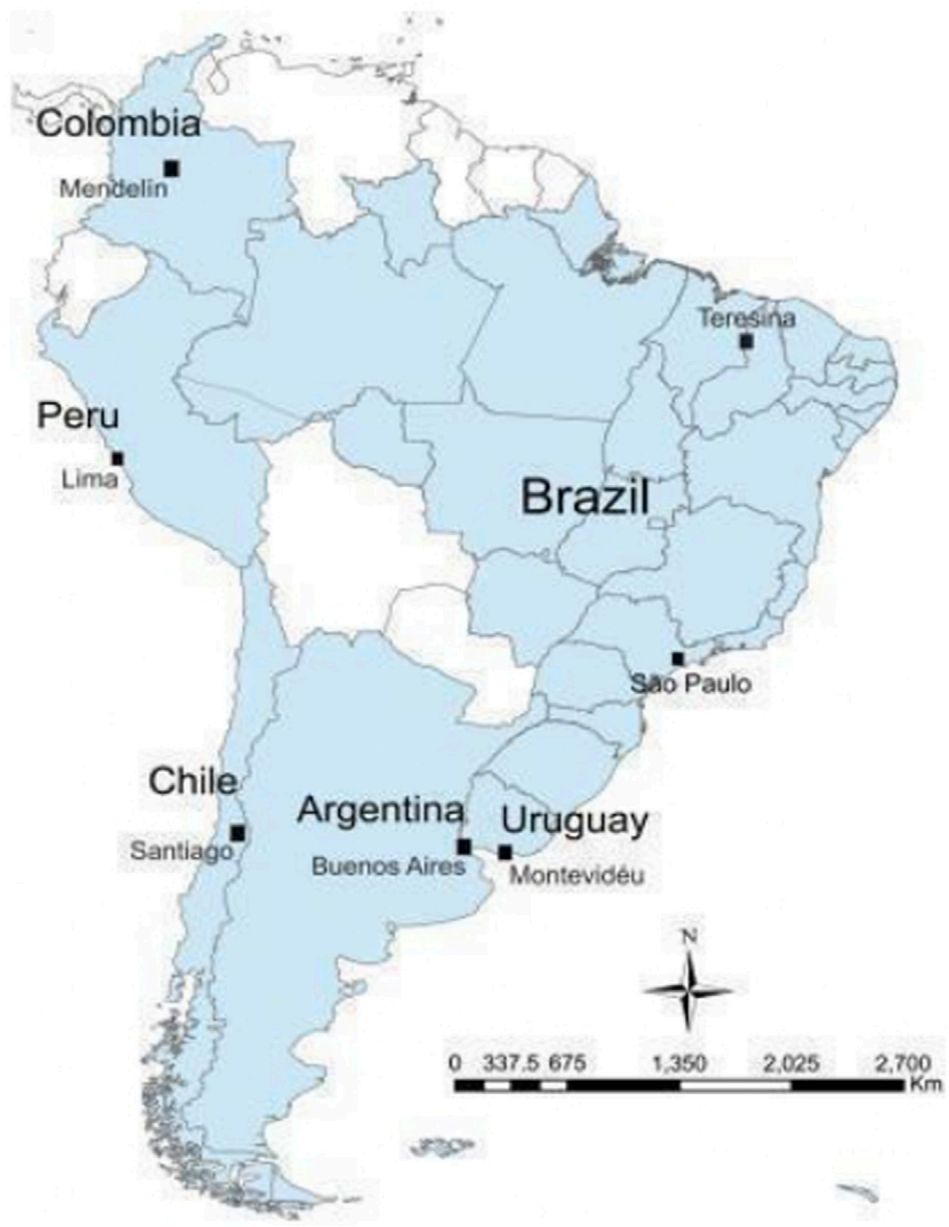
South American Youth/Child Cardiovascular and Environmental (SAYCARE) Study (2015/2016) research centers by country. *SAYCARE Study, South American Youth/Child Cardiovascular and Environmental Study held in Buenos Aires (Argentina), Lima (Peru), Medellin (Colombia), Montevideo (Uruguay), Santiago (Chile), and São Paulo and Teresina (Brazil).

This study utilized data from the “South American Youth/Child Cardiovascular and Environmental (SAYCARE)” Study, an observational, cross-sectional epidemiological investigation conducted across seven South American cities: Buenos Aires (Argentina), Lima (Peru), Medellín (Colombia), Montevideo (Uruguay), Santiago (Chile), São Paulo (Brazil), and Teresina (Brazil) in the academic year 2015 and 2016. These cities were selected based on their hosting of specialized research centers and their populations exceeding 500,000 inhabitants (Figure 1).

### Study Population

The general study population consisted of 1,067 children (aged 3–10 years) and 495 adolescents (aged 11–18 years), enrolled in educational institutions ranging from preschool to the third year of high school, encompassing both public and private schools across the participating cities. From this overall cohort, 351 children and 307 adolescents were selected specifically for the hypertension prediction analyses. The sample size was determined based on prior experience with multicentric projects and insights gained from foundational studies, including the Healthy Lifestyle in Europe by Nutrition in Adolescence Cross-Sectional Study (HELENA-CSS) and the IDEFICS (Identification and Prevention of Dietary- and Lifestyle-Induced Health Effects in Children and Infants) Study [17, 18]. Additionally, a feasibility pilot study was conducted to assess the reliability and validity of the employed methods, ensuring robust methodological underpinnings. This research aims to address critical knowledge gaps in the health of children and adolescents, thereby providing a solid evidence base for future health interventions and policy initiatives targeting these populations [19].

### Blood Pressure Measurement

Blood pressure was measured using the Omron HEM-7200, a validated digital oscillometric device for pediatric populations [20]. Calibration involved activating the inflation mechanism and was performed for all devices used during the study. Measurements were taken on the right arm to account for potential aortic coarctation, with the arm positioned at heart level. Participants sat in a quiet setting with their backs supported, one arm resting on a flat surface, and feet uncrossed and flat on the floor. After a 5-min rest, blood pressure was measured following protocols from the American Heart Association and British Hypertension Society [21]. Two readings were taken 2 min apart; a third measurement was conducted if the difference between readings exceeded 5 mmHg. Elevated blood pressure was defined as systolic or diastolic readings above the 95th percentile for sex, age, and height, per American Academy of Pediatrics guidelines. Sensitivity and specificity analyses assessed the accuracy of the Omron HEM-7200 compared to mercury column readings [22].

### Data Preprocessing

#### Missing Data Imputation and Preprocessing

The treatment of missing data prioritized dataset integrity and minimization of bias, following a structured and systematic approach. Initially, variables not selected for inclusion in the study were discarded. Next, missing values were identified, and their prevalence was quantified for each variable.

Records with missing values in critical variables (i.e., those with more than 30% missing data) were removed to prevent significant analytical bias. For variables with less than 30% missing values, imputation was performed using the median for numerical variables and the mode for categorical variables, ensuring that essential information was retained while maintaining dataset consistency.

To further enhance data quality and model performance, highly correlated variables (correlation coefficient >0.90) were eliminated to mitigate multicollinearity issues. The final dataset underwent rigorous validation to confirm its suitability for subsequent predictive modeling tasks.

This approach, grounded in established best practices, ensured a robust, reliable, and analytically sound dataset while minimizing unnecessary data loss or distortion.

#### Predictors Variables

The variable selection process was guided by evidence from previous studies, a comprehensive literature review, and consultations with subject matter experts [23, 24]. The predictors were measured using reliable and validated questionnaires tailored to the age group, derived from the SAYCARE Study [20, 25–31], whose instruments underwent rigorous validation and adaptation processes to ensure their accuracy and suitability.

A total of 28 variables were incorporated into the predictive model including sociodemographic [biological sex, age], socioeconomic factors [household income (monthly family income based on the minimum wage) and maternal education (< high school, high school, technical education, ≥ university degree)], environmental [sex, age, place of residence, the specific location of the school and questions about the social environment and infrastructure of the residential area], and energy balance behaviors [dietary intake patterns (food items usual consumption), daily physical activity level (moderate-to-vigorous physical activity during physical education classes, leisure time, and transportation), sleep duration (average hours total night sleep time), and daily screen time (spends in front of a television or computer or playing video games)] and waist circumference (cm). The entire description of the predictors is in the Supplementary Material S1, S2.

#### Ethical Considerations

The study followed strict ethical guidelines to ensure participant safety and informed consent. A formal request detailing the study’s objectives and methodology was presented to school administrations, allowing them to consent to participate in the project. For the schools that agreed, potential participants and their parents or guardians received an information letter and a verbal explanation. Informed consent was obtained through the signed Informed Consent Form (ICF) by parents or guardians, and participants provided their signatures on an Assent Form when required. The study was approved by the Research Ethics Committee of the Faculty of Medicine of the University of São Paulo (FMUSP) under research protocol no 232/14, as well as by the respective research ethics committees of all participating centers.

### Statistical Analysis

Descriptive analyses were performed using mean and standard deviation (SD) for continuous variables and percentages for categorical variables. The machine learning methods used in this study included Random Forest, XGBoost, LightGBM, and CatBoost, chosen for their robustness, ability to handle heterogeneous data, and high effectiveness in modeling nonlinear relationships. The analyses were conducted using Python (version 3.6.5), with the support of libraries such as scikit-learn and SHAP for interpretability analysis.

To evaluate the model’s performance, we employed metrics such as accuracy, recall, F1 score, and area under the ROC curve (AUC-ROC), with AUC-ROC serving as the primary criterion for selecting the final model, complemented by consistent results in other metrics. A 5-fold cross-validation was performed to ensure that the models were tested on different data subsets, reducing the risk of overfitting.

Hyperparameter optimization was carried out using the GridSearchCV function, enabling a systematic search for the best parameter combinations to maximize performance. This methodological approach ensured the robustness and reliability of the predictive models, contributing to an accurate and consistent analysis of hypertension in the pediatric population.

#### Model Development and Performance

The study population was randomly divided into training and test sets, comprising 70% and 30% of the total sample, respectively. Hyperparameter tuning was performed to enhance model performance, with the GridSearchCV function from the scikit-learn package utilized to identify the optimal hyperparameters.

A 5-fold cross-validation was employed during training to evaluate the model’s performance and mitigate the risk of overfitting. The final assessment of model performance was conducted exclusively on the test set. Feature importance rankings were calculated based on the differences in the approaches used by each model.

Four widely recognized algorithms for supervised predictive analysis were employed in this study: Random Forest, XGBoost, LightGBM, and CatBoost. Model performance was evaluated using standard predictive metrics, including accuracy, recall, F1 score, and the AUC-ROC. Model selection prioritized the algorithm with the highest AUC, alongside consistent performance across the other metrics.

#### Class Imbalance

To address class imbalance in the training dataset, an oversampling strategy was employed. An initial assessment of the class distribution revealed a significant discrepancy between the majority class (class 0: non-hypertensive) and the minority class (class 1: hypertensive). To mitigate this issue, the RandomOverSampler function was used to apply an oversampling technique, setting the oversampling ratio to 1. This process resulted in an equal number of samples for both classes. This approach was essential to ensure balanced representation of both classes during model training, thereby improving the performance and reliability of the predictive models for hypertension.

### Transfer Learning

Transfer Learning (TL) is a machine learning approach that leverages knowledge gained in a specific domain or task and applies it to another, related domain or task. This technique has been widely utilized in various fields, including image recognition and classification, to improve model performance and efficiency when data in the target domain is limited or less informative [32, 33].

In this study, TL was employed to enhance the performance of machine learning models in the early detection of hypertension in pediatric populations, specifically children and adolescents. The TL technique used involves decision tree-based algorithms, where the trees learned from an initial model (e.g., predicting hypertension in children) are transferred to a similar algorithm applied to a different, less robust dataset (e.g., adolescents). This incremental learning process improves model performance on the adolescent sample, which would otherwise yield suboptimal results if trained exclusively on its own data.

Initially, the sample of children was used as the source domain for pre-training the models due to its more consistent and complete feature set, which provided a strong foundation for model training. The knowledge acquired during this phase was then transferred to the adolescent sample, enabling the models to adapt effectively to the nuances of this population. This approach was particularly advantageous, as the adolescent sample exhibited greater variability and a smaller volume of relevant data, making it an ideal target domain for TL. By utilizing this methodology, the study optimized the use of available data, ensuring improved model performance and a more accurate identification of hypertension across distinct age groups [34, 35].

#### Model Explanation and Individual Analysis

The SHapley Additive exPlanations (SHAP) method provides an interpretable approach to understanding machine learning (ML) models. This model-agnostic technique evaluates the local contribution of each variable while offering a global perspective on model performance, including metrics such as accuracy, relevance, and local consistency. In this study, the SHAP algorithm was employed to investigate the contribution and importance of individual features and to analyze the nonlinear interactions between risk predictors [36].

## Results

Table 1 summarizes the demographic, metabolic, and dietary characteristics of the children and adolescents included in the study. Most participants attended public schools, with a balanced distribution of sexes in both age groups. Children and adolescents showed differences in mean age and waist circumference, with adolescents displaying greater variability. Dietary habits varied widely, particularly for items such as soft drinks, filled cookies, and chips, reflecting diverse consumption patterns. Adolescents generally reported higher intake of most food items than children, potentially due to differing dietary preferences or caloric needs.

**TABLE 1:** Descriptive statistics of the features included in the predictive model, categorized by age group, South American Youth/Child Cardiovascular and Environmental (SAYCARE) Study (2015/2016).

Feature	Children	Adolescents
Sex [n (%)]		
Female	246 (52.6)	157 (51.1)
Male	222 (47.4)	150 (48.9)
School [n (%)]		
Public	265 (56.6)	176 (57.3)
Private	203 (43.4)	131 (42.7)
Family Income [n (%)]		
1 Minimum Wage	6 (6.7)	18 (11.8)
1 to 2 Minimum Wage	12 (13.3)	36 (23.7)
2 to 5 Minimum Wage	12 (13.3)	37 (24.3)
5 to 10 Minimum Wage	14 (15.6)	17 (11.2)
10 to 15 Minimum Wage	9 (10.0)	10 (6.6)
15 to 20 Minimum Wage	6 (6.7)	6 (3.9)
20 to 25 Minimum Wage	5 (5.6)	2 (1.3)
More than 25 Minimum Wage	7 (7.8)	6 (4.0)
Don’t know/Will not inform	19 (21.1)	20 (13.2)
Maternal Education Level [n (%)]		
Lower education	7 (7.3)	16 (10.1)
Lower secondary education	11 (11.4)	11 (7.0)
Higher secondary education	17 (17.7)	47 (29.7)
University degree	50 (52.1)	61 (38.6)
Technical education	11 (11.4)	21 (13.3)
Without education	0 (0.0)	2 (1.3)
Availability of fruits and vegetables at home [n (%)]		
Always	168 (58.1)	109 (53.2)
Almost always	85 (29.4)	57 (27.8)
Sometimes	26 (9.0)	29 (14.1)
Rarely	8 (2.8)	7 (3.4)
Never	2 (0.7)	3 (1.5)
Availability of dairy products at home [n (%)]		
Always	194 (68.6)	120 (60.0)
Almost always	51 (18.0)	47 (23.5)
Sometimes	21 (7.4)	23 (11.5)
Rarely	13 (4.6)	5 (2.5)
Never	4 (1.4)	5 (2.5)
Availability of breads/cereals at home [n (%)]		
Always	157 (55.5)	94 (47.7)
Almost always	69 (24.4)	57 (28.9)
Sometimes	38 (13.4)	35 (17.8)
Rarely	12 (4.2)	9 (4.6)
Never	7 (2.5)	2 (1.0)
Adequacy of sweets/snacks consumption [n (%)]		
Always	7 (2.5)	14 (7.2)
Almost always	7 (2.5)	19 (9.7)
Sometimes	88 (31.7)	62 (31.8)
Rarely	90 (32.4)	63 (32.3)
Never	86 (30.9)	37 (19.0)
Availability of sweets/snacks at home [n (%)]		
Always	12 (4.3)	16 (8.4)
Almost always	27 (9.6)	28 (14.7)
Sometimes	72 (25.5)	58 (30.4)
Rarely	99 (35.1)	59 (30.9)
Never	72 (25.5)	30 (15.7)
Permission to watch TV during meals [n (%)]		
Always	26 (9.2)	69 (34.3)
Almost always	32 (11.3)	39 (19.4)
Sometimes	101 (35.8)	47 (23.4)
Rarely	58 (20.6)	19 (9.5)
Never	65 (23.0)	27 (13.4)
Consumption of fruits/vegetables as a snack without asking permission [n (%)]		
Always	122 (43.4)	127 (64.5)
Almost always	49 (17.4)	34 (17.2)
Sometimes	47 (16.7)	13 (6.6)
Rarely	39 (13.9)	9 (4.6)
Never	24 (8.5)	14 (7.1)
Consumption of breads/cereals as a snack without asking permission [n (%)]		
Always	71 (25.5)	110 (55.8)
Almost always	32 (11.5)	36 (18.3)
Sometimes	58 (20.9)	17 (8.6)
Rarely	46 (16.6)	15 (7.6)
Never	71 (25.5)	19 (9.7)
Snacks or sweets as a reward or consolation [n (%)]		
Always	6 (2.1)	8 (4.0)
Almost always	5 (1.8)	4 (2.0)
Sometimes	52 (18.2)	22 (11.1)
Rarely	67 (23.5)	35 (17.7)
Never	155 (54.4)	129 (65.2)
Strict food rules [n (%)]		
Always	85 (30.2)	21 (10.7)
Almost always	75 (26.7)	16 (8.2)
Sometimes	68 (24.2)	37 (18.9)
Rarely	28 (10.0)	35 (17.8)
Never	25 (8.9)	87 (44.4)
Parents' consumption of sweets/snacks in front of children/adolescents [n (%)]		
Always	51 (18.3)	29 (14.9)
Almost always	33 (11.9)	21 (10.8)
Sometimes	71 (25.5)	53 (27.3)
Rarely	49 (17.6)	46 (23.7)
Never	33 (11.9)	45 (23.2)
Satisfaction with snack consumption habits [n (%)]		
Always	81 (29.2)	60 (30.9)
Almost always	70 (25.3)	42 (21.7)
Sometimes	64 (23.1)	51 (26.3)
Rarely	35 (12.6)	21 (10.8)
Never	27 (9.7)	20 (10.3)
Pleasant mealtime moments [n (%)]		
Always	171 (60.9)	90 (46.1)
Almost always	83 (29.5)	57 (29.2)
Sometimes	20 (7.1)	38 (19.5)
Rarely	5 (1.8)	5 (2.6)
Never	2 (0.7)	5 (2.6)
Parent-child relationship [n (%)]		
Always	242 (86.1)	82 (44.8)
Almost always	32 (11.4)	47 (25.7)
Sometimes	6 (2.1)	34 (18.6)
Rarely	0 (0.0)	14 (7.7)
Never	1 (0.4)	6 (3.3)
Happiness at home [n (%)]		
Always	231 (83.7)	124 (63.3)
Almost always	32 (11.6)	37 (18.9)
Sometimes	7 (2.5)	25 (12.8)
Rarely	2 (0.7)	5 (2.5)
Never	4 (1.5)	5 (2.5)
Arguments at home in front of the child/adolescent [n (%)]		
Always	5 (1.8)	10 (5.1)
Almost always	6 (2.2)	20 (10.2)
Sometimes	67 (24.3)	44 (22.3)
Rarely	112 (40.6)	38 (19.2)
Never	86 (31.1)	38 (19.2)
Overprotection of the child/adolescent [n (%)]		
Always	56 (20.0)	65 (32.8)
Almost always	31 (11.1)	17 (8.6)
Sometimes	86 (30.7)	40 (20.2)
Rarely	62 (22.1)	38 (19.2)
Never	45 (16.1)	38 (19.2)
Screen time [n (%)]		
<2 h/day	38 (23.5)	53 (20.6)
>2 h/day	124 (76.5)	204 (79.4)
High Blood Pressure [n (%)]	14.1 (50.0)	7.5 (23.0)
Age (mean [±std)]	6.9 (2.3)	14.7 (2.1)
Mean waist circumference [mean (±std)]	59.0 (9.9)	73.5 (9.2)
Sleep duration [mean (±std)]	9.2 (0.9)	8.1 (1.6)
Total Physical Activity [mean (±std)]	65.2 (105.9)	41.9 (48.2)
Duration of exclusive breastfeeding [mean (±std)]	7.9 (11.7)	13.6 (20.4)
Daily fruits consumption in grams [mean (±std)]	08.7 (321.1)	203.4 (388.9)
Daily vegetables consumption in grams [mean (±std)]	58.5 (237.6)	56.6 (130.5)
Daily crackers consumption in grams [mean (±std)]	20.8 (72.9)	30.0 (71.7)
Daily cookies consumption in grams [mean (±std)]	12.7 (51.5)	20.9 (66.1)
Daily filled cookie consumption in grams [mean (±std)]	14.5 (71.6)	42.2 (126.9)
Daily baked goods consumption in grams [mean (±std)]	13.0 (89.1)	23.1 (85.9)
Daily pizza consumption in grams [mean (±std)]	6.4 (17.7)	26.2 (108.9)
Daily hamburger consumption in grams [mean (±std)]	5.5 (14.0)	19.2 (47.9)
Daily breaded meat consumption in grams [mean (±std)]	14.0 (86.1)	40.3 (163.0)
Daily sausage consumption in grams [mean (±std)]	8.8 (59.2)	11.79 (34.9)
Daily cold meat consumption in grams [mean (±std)]	5.9 (27.0)	12.5 (37.6)
Daily fish consumption in grams [mean (±std)]	7.9 (21.5)	18.6 (84.3)
Daily soft drink consumption in grams [mean (±std)]	52.2 (155.0)	128.7 (241.3)
Daily chips consumption in grams [mean (±std)]	5.8 (27.4)	8.8 (34.5)
Daily mayonnaise consumption in grams [mean (±std)]	4.1 (15.4)	8.3 (23.8)
Daily sauces consumption in grams [mean (±std)]	3.7 (9.9)	7.8 (17.4)
Daily finger foods consumption in grams [mean (±std)]	1.8 (8.4)	8.2 (55.9)

Table 2 compares the performance of different machine learning models for diagnosing hypertension in children and adolescents. For children, all models achieved accuracies close to 90% before the application of transfer learning (TL). The Random Forest model demonstrated the highest precision (0.83), while XGBoost and LightGBM showed slightly lower values (around 0.72). In terms of recall, XGBoost and LightGBM performed best, with values of 0.50, followed by Random Forest and CatBoost, both at 0.31. The models exhibited comparable AUC-ROC, ranging between 0.71 and 0.74.

**TABLE 2:** Performance comparison of machine learning models for children and adolescents without transfer learning (South America, 2015-2016).

Children					
	Model	Accuracy	Precision	Recall	AUC(ROC)
	Random Forest	0.90	0.83	0.31	0.71
	XGBoost	0.90	0.72	0.50	0.74
	LightGBM	0.90	0.72	0.50	0.74
	Catboost	0.88	0.71	0.31	0.73

Adolescents					
	Model	Accuracy	Precision	Recall	AUC(ROC)
	Random Forest	0.86	0.00	0.00	0.46
	XGBoost	0.85	0.00	0.00	0.51
	LightGBM	0.86	0.00	0.00	0.53
	Catboost	0.86	0.00	0.00	0.56

For adolescents, the models also achieved high accuracy, with scores around 0.85 and 0.86. However, their discriminative ability, as measured by the AUC-ROC, was relatively low. CatBoost achieved the highest AUC-ROC value at 0.56, followed by LightGBM (0.53) and XGBoost (0.51). Random Forest showed the lowest discriminative ability, with an AUC-ROC of 0.46.

Table 3 presents the results after applying transfer learning to the models. All models exhibited consistent accuracy at 0.86, indicating strong overall performance in data classification. Notably, both LightGBM and CatBoost achieved a precision of 1.0, reflecting their enhanced ability to correctly identify positive cases and modest improvements compared to previous metrics. Regarding discriminative ability, as measured by the AUC-ROC, CatBoost achieved the highest score of 0.82, demonstrating superior capacity to distinguish between positive and negative cases after transfer learning.

**TABLE 3:** Performance comparison of machine learning models for adolescents after applying transfer learning (South America, 2015-2016).

Adolescents					
	Model	Accuracy	Precision	Recall	AUC(ROC)
	XGBoost	0.86	0.50	0.06	0.77
	LightGBM	0.86	1.0	0.06	0.72
	Catboost	0.86	1.0	0.18	0.82

**FIGURE 2:**
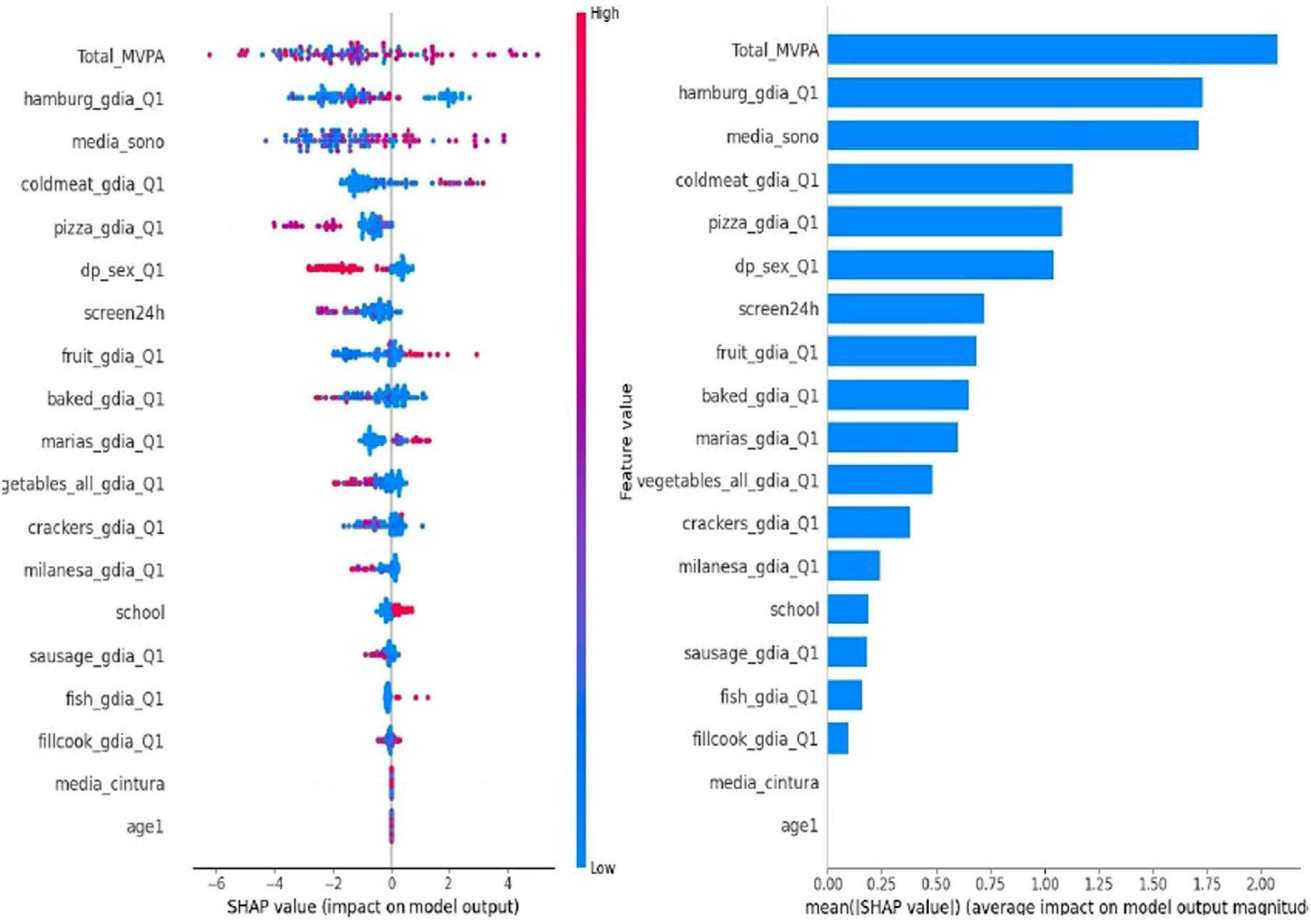
SHapley Additive exPlanations (SHAP) Analysis {[Buenos Aires (Argentina), Lima (Peru), Medellin (Colombia), Montevideo (Uruguay), Santiago (Chile), and São Paulo and Teresina (Brazil). 2015/2016]}.

The SHAP plot (Figure 2A) provides a detailed analysis of the variables influencing hypertension prediction in the pediatric population. Key contributors to hypertension prediction included low physical activity, increased screen time, shorter sleep duration, higher waist circumference, and greater consumption of foods such as hamburgers, cold cuts, pizza, and sausages. Figure 2B illustrates the mean importance of these variables, offering a comprehensive view of their relative impact on the model. These findings provide valuable insights for the development of targeted preventive strategies and interventions to address hypertension in this age group.

## Discussion

In this multicentric observational study, algorithms were developed and evaluated to predict the presence of hypertension in the pediatric population across seven South American cities. The results demonstrate an improvement in predictive performance with the application of transfer learning (TL) in this population.

Hypertension prevention strategies can target the general population or specific high-risk groups. The increasing demand for early identification of at-risk individuals who could benefit from preventive interventions has driven interest in predictive models for hypertension [23]. While numerous models have been developed for the adult population using both traditional regression-based approaches and machine learning methods, there is a notable gap in pediatric-focused models [24, 32], because Pediatric hypertension affects approximately 11% of boys and 9.6% of girls globally, presenting a significant public health concern that the American Heart Association has recently highlighted as critical to address [37, 38].

This study builds on prior research by applying TL to predict hypertension in the pediatric population, proving it to be an effective strategy. A substantial sample of children was used to pre-train a model, which was then fine-tuned using the adolescent sample [34]. This approach leveraged general features from the larger and more robust dataset to adapt to the specific characteristics of adolescents. The results revealed a significant improvement in the AUC-ROC, highlighting the model’s enhanced ability to differentiate adolescents at risk for hypertension. These findings underscore the potential of TL in data-scarce contexts, maximizing the utility of available information and improving generalization and accuracy in predictions [35].

TL is a cornerstone of artificial intelligence (AI), enabling the reuse of pre-trained models for new tasks, thereby saving time and computational resources. This technique is particularly valuable in domains where acquiring large volumes of labeled data is challenging or costly. Studies have demonstrated that TL accelerates the development of AI solutions by leveraging knowledge from previously learned tasks, enhancing model efficiency and effectiveness [39]. Furthermore, TL democratizes AI by making advanced solutions accessible to organizations with limited resources, reducing the dependency on extensive datasets or advanced computing infrastructure. In healthcare, TL significantly enhances the predictive capabilities of models and broadens the applicability of AI technologies for early detection and disease management [3].

Although the application of SHAP in predicting hypertension in the pediatric population is still limited, recent studies have explored its use in related contexts. One study utilized SHAP to interpret machine learning models for hypertension risk prediction, identifying significant risk factors such as elevated LDL cholesterol levels and low HDL cholesterol levels [40, 41].

Our findings, corroborated by SHAP analysis, are consistent with existing literature linking sedentary lifestyles and diets high in processed foods to an increased risk of hypertension. The results emphasize the importance of interventions focused on promoting physical activity and healthy eating habits from childhood as crucial strategies for preventing hypertension and promoting long-term cardiovascular health. The inclusion of public policies and educational programs aimed at reducing screen time and improving sleep quality can also play a vital role in mitigating these risk factors [42].

Working with pediatric data to predict arterial hypertension presents several limitations that may impact the accuracy and applicability of predictive models. First, the inherent biological variability in growth and development during childhood leads to significant variations in physiological parameters, including blood pressure [43]. This variability makes the creation of robust and consistent predictive models a substantial challenge. Additionally, the definition of hypertension in children is based on age-, sex-, and height-adjusted percentiles, which adds a layer of complexity to standardizing diagnostic criteria and comparing different studies [36, 44]. Another limitation is data availability. Compared to adults, there is a significantly smaller amount of data on childhood hypertension, making it difficult to identify robust patterns and validate predictive models.

This study has certain limitations that should be acknowledged. The relatively small sample size, particularly for the adolescent group, may impact the generalizability and robustness of the findings [4]. To address these limitations, a feasibility pilot study was conducted to validate the reliability of the methods, and statistical adjustments were made to account for the sample structure. Despite these constraints, the study provides valuable insights into the early detection of hypertension in pediatric populations and highlights the need for future research with larger, more diverse samples to validate and extend the present findings.

Therefore, it is essential to continue expanding pediatric databases, improve data collection methods, standardize diagnostic criteria, and develop algorithms that consider the variability and particularities of the pediatric population to overcome these limitations and enhance the accuracy of predictive models [45, 46].

These findings have significant implications for developing intervention strategies and health policies to prevent and manage childhood hypertension. They highlight the potential of AI-based modeling approaches to identify and analyze risk factors in public health. Moreover, the results underscore the importance of individualized health promotion strategies that account for the diverse needs and behaviors of pediatric populations.

### Conclusion

Machine learning models effectively identified key dietary predictors of elevated blood pressure in children and adolescents. High consumption of soft drinks, filled cookies, and chips were identified as significant risk factors, with adolescents exhibiting a higher intake of these foods compared to children. These findings emphasize the critical need to address unhealthy dietary habits through early prevention strategies aimed at reducing the risk of hypertension and fostering cardiovascular health in pediatric populations.
