# Supplementary material for: Prediction of Hypertension in the Pediatric Population Using Machine Learning and Transfer Learning: A Multicentric Analysis of the SAYCARE Study
Source: Int J Public Health. 2025 Mar 11;70:1607944. doi: 10.3389/ijph.2025.1607944 (PMC11937837; doi:10.3389/ijph.2025.1607944)
Supplement: Supplementary file 1 [file DataSheet1.PDF]

Supplement 1

Predictor variables used in the analysis, with codes and descriptions provided

Categorical

Code	**Description**
Dp_sex_Q1	Gender
School	Type of School
Fs	Maternal Education
Family_income	Family Income
Dd171_Q1	Availability of Fruits and Vegetables at Home
Dd172_Q1	Availability of Dairy Products at Home
Dd173_Q1	Availability of Bread/Cereals at Home
Dd174_Q1	Is Consumption of Sweets/Snacks Adequate?
Dd175_Q1	Availability of Sweets/Snacks at Home
Dd176_Q1	Permission to Watch TV During Meals
Dd177_Q1	Consumption of Fruits/Vegetables as Snacks Without Asking
Dd178_Q1	Consumption of Bread/Cereals as Snacks Without Asking
Dd179_Q1	Consumption of Sweets/Snacks as a Reward or Comfort
Dd1710_Q1	Strict Food Rules
Dd1711_Q1	Parental Consumption of Sweets/Snacks in Front of Children
Dd1712_Q1	Satisfaction with Snacking Habits
Dd1713_Q1	Pleasant Eating Environment
Dd1714_Q1	Good Parent-Child Relationship
Dd1716_Q1	Child's Happiness at Home
Dd1717_Q1	Overprotection of the Child
Screen24h	Screen Time

Continuous Variables

Code	**Description**
Age1	Age
Media_cintura	Waist Circumference
Total_MVPA	Physical Activity
Media_sono	Sleep
Fm	Duration of Exclusive Breastfeeding
Fruit_gdia_Q1	Fruits Consumed Per Day (g)
Vegetables_all_gdia_Q1	Vegetables Consumed Per Day (g)
Crackers_gdia_Q1	Crackers Consumed Per Day (g)
Marias_gdia_Q1	Sweet Biscuits Consumed Per Day (g)
Fillcook_gdia_Q1	Filled Cookies Consumed Per Day (g)
Baked_gdia_Q1	Bakery Products Consumed Per Day (g)
Pizza_gdia_Q1	Pizza Consumed Per Day (g)
Hamburg_gdia_Q1	Hamburgers Consumed Per Day (g)
Milanesa_gdia_Q1	Breaded Fillets Consumed Per Day (g)
Sausage_gdia_Q1	Sausages Consumed Per Day (g)
Coldmeat_gdia_Q1	Cold Meat Consumed Per Day (g)
Fish_gdia_Q1	Fish Consumed Per Day (g)
Softdrnk_gdia_Q1	Soft Drinks Consumed Per Day (g)
Chips_gdia_Q1	Chips Consumed Per Day (g)
Mayonn_gdia_Q1	Mayonnaise Consumed Per Day (g)
Sauces_gdia_Q1	Sauces Consumed Per Day (g)
Spsalgad_gdia_Q1	Fried Snacks Consumed Per Day (g)

Supplement 2: Method of measuring predictor variables

Demographic and Environmental Factors Questionnaire

The study included questions about sex, age, place of residence, and the specific location of the school, along with inquiries about the social environment and residential area infrastructure. Environmental characteristics were evaluated using a 15-item questionnaire that addressed factors such as the quality of public spaces, access to physical activity environments, perceptions of local violence, garbage collection frequency, and other cardiovascular risk-related factors. The environmental questionnaire demonstrated good reproducibility across both age groups (k = 0.612 for children and k = 0.746 for adolescents)[1,2].

Socioeconomic Factors Questionnaire

Family income ($)

Monthly family income was collected in five categories based on the minimum wage in the country at the start of data collection. Similarly, the SES questionnaire exhibited strong reliability for all indicators in both age groups (k = 0.991 for children and k = 0.964 for adolescents)[3].

Household Characteristics

The questionnaire was adapted to the Brazilian context using the socioeconomic classification criteria of the Brazilian Association of Research Companies (ABEP). These criteria consider the presence and types of assets or household characteristics, such as the existence of individualrooms for the child, the number of bathrooms with a shower and toilet, the number of televisions, vehicles, and the ownership of goods like radios, refrigerators, DVD or VCR players, telephone lines, and computers. Additionally, questions were included about access tolaptops, internet (broadband or dial-up), video games (traditional and/or mobile), and domesticworkers [4]. The total score showed a moderate to strong internal consistency coefficient for age groups (Cronbach's α coefficients = 0.907 for children and Cronbach's α coefficients = 0.593 for adolescents)[4].

Maternal Education

Classified as high school or less, some college but no degree, or bachelor's degree or higher, according to years of formal education in school. The total score showed a strong reliability coefficient for age groups (Kappa coefficients = 0.955 for children and Kappa coefficients = 0.964 for adolescents)[5].

Behaviors related to energy balance

Physical activity

Accelerometer

The ActiGraph MTI accelerometer (GT3X model) was used to objectively measure physical activity. Participants wore the device on their waist for at least eight hours daily over seven consecutive days. Parents/guardians and children were instructed to wear the device during waking hours, avoiding water-based activities, and to remove it at night. Daily logs were provided for recording device use, removal/replacement instances, activities performed, and timings. Periods with zero counts per minute (CPM) exceeding 20 minutes or exceeding 20,000 CPM were excluded from analysis[6,7].

Valid data was defined as at least six hours of use per day on a minimum of three days. Physical activity data were analyzed as CPM/day, with activity intensity categorized as light (26–573 CPM), moderate (574–1,002 CPM), or vigorous (≥1,003 CPM) based on validated cutoff points for each age group. Participants were classified as active if they achieved an average of at least 60 minutes per day of moderate-to-vigorous physical activity, in line with current guidelines.

In children, the physical activity questionnaire demonstrated consistent reliability (ρ = 0.56) and moderate validity (ρ = 0.46), with contextual variables explaining 43.0% of the variance and a bias of −22.9 min/day. For adolescents, reliability was higher (ρ = 0.76), and validity was near excellent (ρ = 0.88), with 66.7% of the variance explained at the city level and a bias of 16.0 min/day[8,9].

Questionnaire

The questionnaire assessed routine activities over the past week across three domains: school, leisure, and commuting. It calculated leisure physical activity time by averaging weekday and weekend data, weighted accordingly. Physical activity intensity was categorized into light, moderate, and vigorous levels based on subjective fatigue.

Time spent in moderate and vigorous activities was analyzed, and participants were classified as active if they accumulated an average of at least 60 minutes/day of moderate-to-vigorous physical activity using the subjective fatigue scale[9].

Sedentary Behavior and Time Questionnaire

Sedentary behavior (SB) was assessed through questions on activities like watching TV, computer use, studying, and gaming, with study time replaced by passive activities such as playing with toys or coloring. Children answered 8 questions, and SB time was calculated by summing the reported daily minutes for weekdays and weekends.

SB was also measured via accelerometers, defining sedentary activity as less than 100 CPM. To standardize data, SB time was adjusted for wear time and recalculated to a 12-hour day

(720 minutes). Total SB time was determined as a weighted average of weekdays and weekends. Participants were classified as meeting or not meeting the recommended limit of less than 120 minutes/day of SB.

The reliability of sedentary behavior time was moderate for children (rho ≥0.45 and k ≥ 0.40) and adolescents (rho ≥0.30)[10].

Sleep Time Questionnaire

The questionnaire addressed sleep time and the routine of the previous week (7 days). Data were collected through questions about sleeping time (duration) and bedtime routine during weekdays and weekends. A total of 8 questions were asked, including information about wake-up time, bedtime, nap time, and nighttime sleep duration. Additionally, the sleep period (night)[11].

The questionnaire demonstrated acceptable temporal stability in children and adolescents for the total number of days (rho≥0.30; p<0.05). For the total number of days, the questionnaire showed acceptable convergent validity in children (rho ranging from 0.48 to 0.62; p≤0.01)[12].

Dietary Determinants

A structured questionnaire with a five-point Likert scale evaluated six factors: body image perception, self-esteem, emotional well-being, food-related behaviors, family and school environment, supplementary feeding, and media influence.

Energy Balance-Related Behaviors

Dietary patterns were assessed using three non-consecutive 24-hour dietary recalls (24HDRs) covering two weekdays and one weekend day. Caregivers provided qualitative and quantitative food intake data using household measures. The first recall was conducted at school with a dietitian, while the remaining recalls were completed at home with the help of a food booklet.

For children and adolescents: reliability [Spearman coefficients ranging from 0.47 to 0.73, intraclass correlation coefficients (ICC) between 0.66 and 0.99, κw coefficients from 0.35 to 0.63, and agreement percentages between 72.75% and 83.52%]; validity [Spearman coefficients ranging from 0.17 to 0.37, energy-adjusted Pearson coefficients between 0.17 and 0.61, κw coefficients from 0.09 to 0.24, and agreement percentages between 45.79% and 67.06%][13].

Supplementary Figure 3. Performance comparison of machine learning models for diseasediagnosis for children

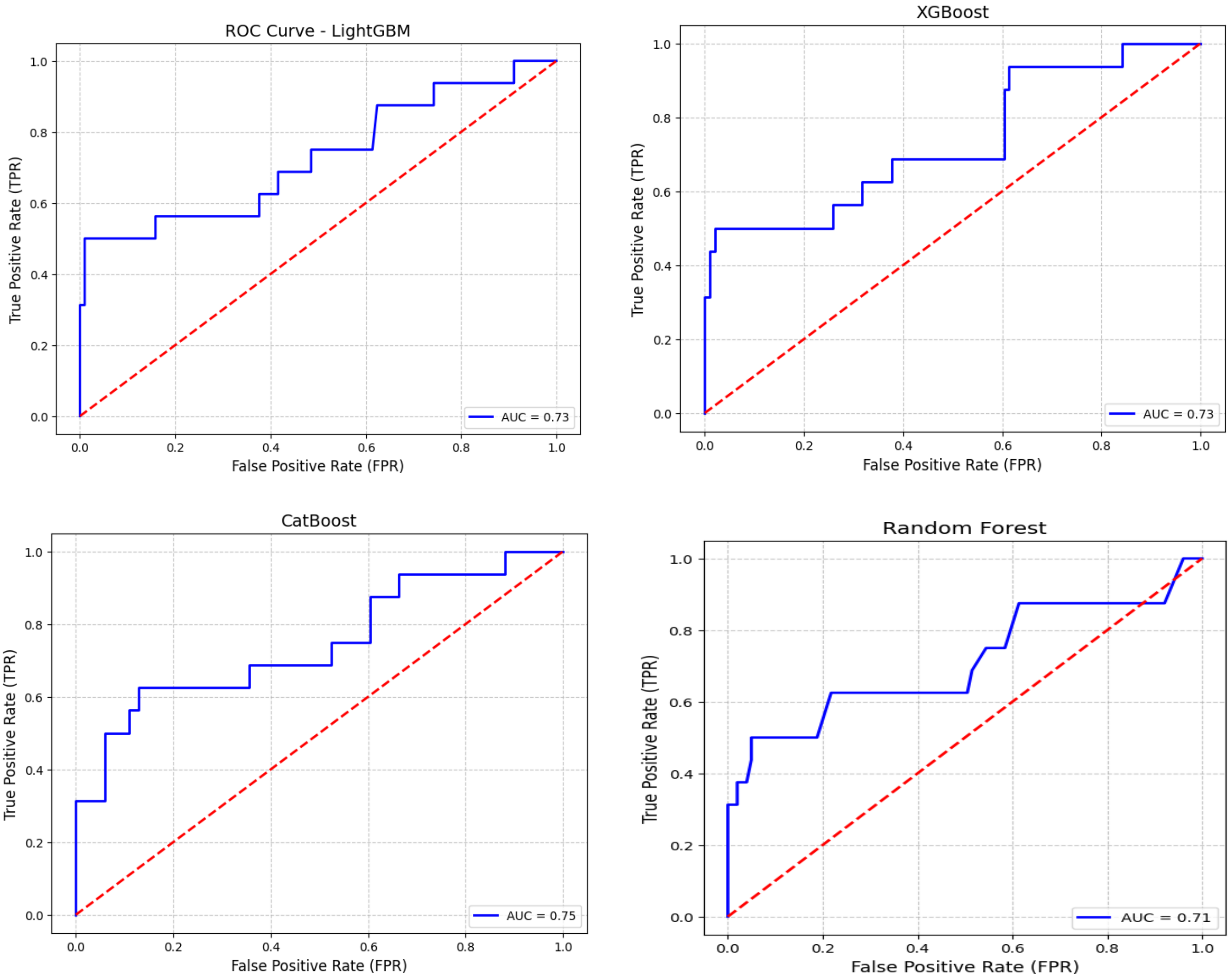

Supplementary Figure 4. Comparison of the performance of machine learning models fordisease diagnosis for adolescents without applying transfer learning

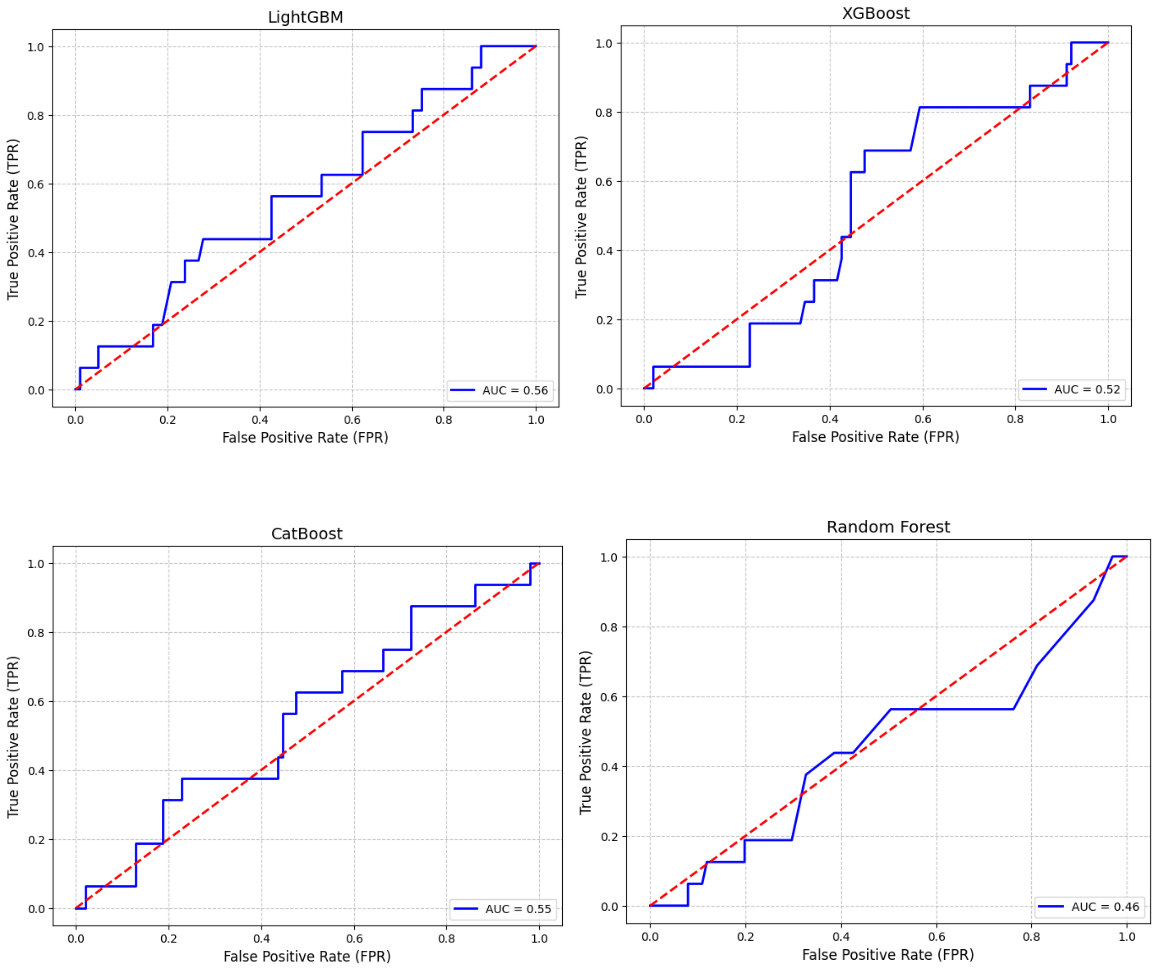

Supplementary Figure 5 a) Correlation matrix between variables for the children model. b) Correlation matrix between variables for the adolescent model

a)

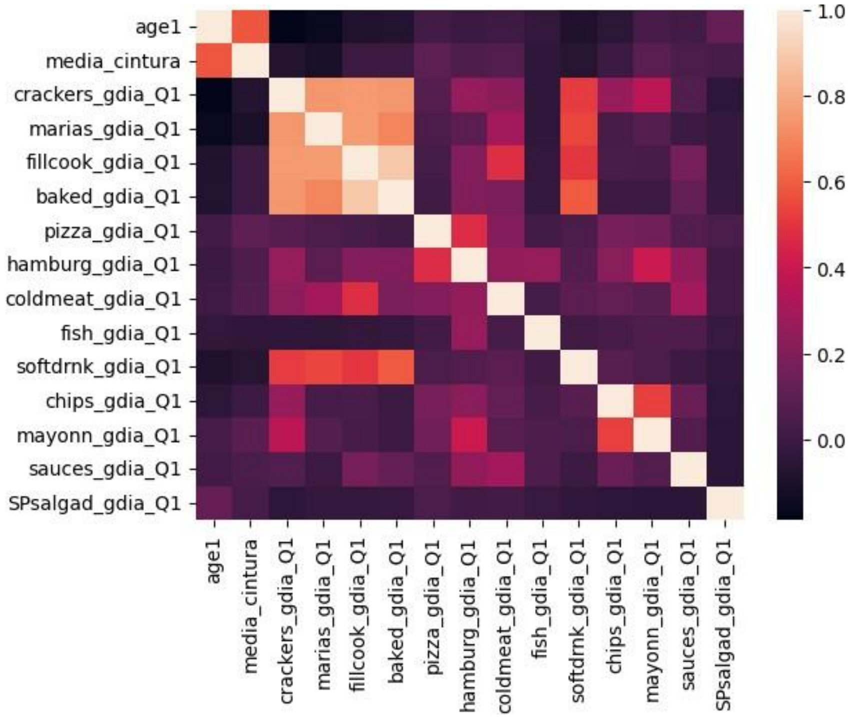

b)

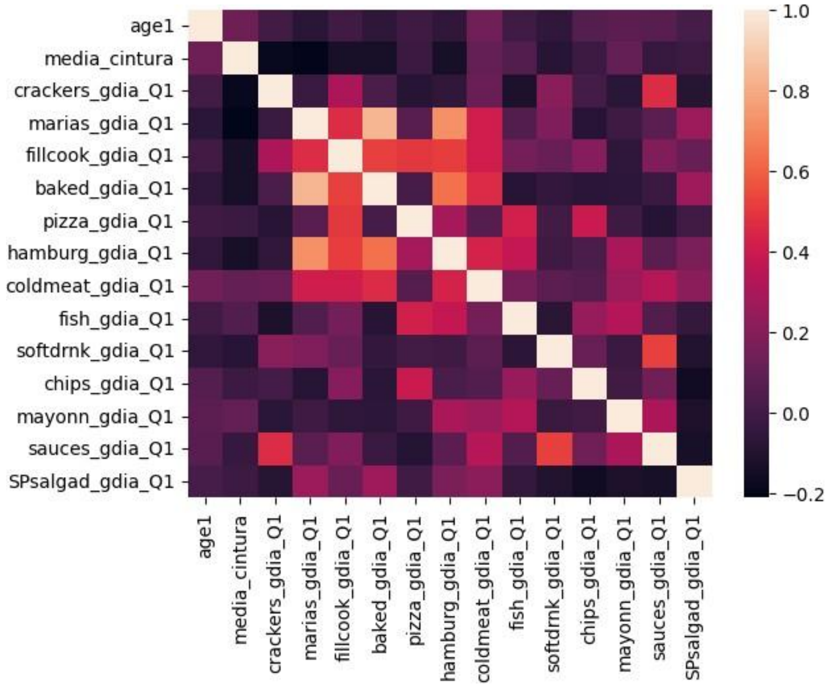

REFERENCES

1. Moraes AC e de Moraes AC; Forkert ECO ; Vilanova-Campelo RC ; González-Zapata L ; Azzaretti L ; Iguacel I ; Huicho L ; Moliterno P ; Moreno LA ; Carvalho HB . Measuring Socioeconomic Status and Environmental Factors in the SAYCARE Study in South America: Reliability of the Methods. Obesity, v. 26, p. S14-S22, 2018.
2. Carvalho HB, Moreno LA, Silva AM, Berg G, Estrada-Restrepo A, González-Zapata LI, De Miguel-Etayo P, Delgado CA, Bove MI, de Sousa MDLR, Torres-Leal FL, Forjaz CLM, De Moraes ACF; SAYCARE Study Group. Design and Objectives of the South American Youth/Child Cardiovascular and Environmental (SAYCARE) Study. Obesity (Silver Spring). 2018 Mar;26 Suppl 1:S5-S13. doi: 10.1002/oby.22117. PMID: 29464921.
3. Lanfer A, Hebestreit A, Ahrens W, Krogh V, Sieri S, Lissner L, *et al.* Reproducibility of food consumption frequencies derived from the Children's Eating Habits Questionnaire used in the IDEFICS study. *Int J Obes (Lond).* 2011; 35 Suppl 1: S61-8.
4. ABEP. [Brazilian association of research institutes]. Brazilian criteria for economic classification. Associação Brasileira de Empresas e Pesquisa. Disponível em: http://www.abep.org/criterio-brasil. 2014.
5. Herrmann D, Suling M, Reisch L, Siani A, De Bourdeaudhuij I, Maes L, *et al.* Repeatability of maternal report on prenatal, perinatal and early postnatal factors: findings from the IDEFICS parental questionnaire. *Int J Obes (Lond).* 2011; 35 Suppl 1: S52-60.
6. Ojiambo R, Cuthill R, Budd H, Konstabel K, Casajús JA, González-Agüero A, *et al.* Impact of methodological decisions on accelerometer outcome variables in young children. *Int J Obes (Lond).* 2011; 35 Suppl 1: S98-103.
7. Ruiz J, Ortega F, Martinez-Gomez D, Labayen I, Moreno L, De Bourdeaudhuij I, *et al.* Objectively Measured Physical Activity and Sedentary Time in European Adolescents: The HELENA Study. *American Journal of Epidemiology.* 2011; 174: 173-84.
8. Bielemann RM, Reichert FF, Paniz VM, Gigante DP. Validation of the Netherlands physical activity questionnaire in Brazilian children. *Int J Behav Nutr Phys Act.* 2011; 8: 45.
9. Nascimento-Ferreira MV, De Moraes ACF, Toazza-Oliveira PV, Forjaz CLM, Aristizabal JC, Santaliesra-Pasías AM, Lepera C, Nascimento-Junior WV, Skapino E, Delgado CA, Moreno LA, Carvalho HB. Reliability and Validity of a Questionnaire for Physical Activity Assessment in South American Children and Adolescents: The SAYCARE Study. Obesity (Silver Spring). 2018 Mar;26 Suppl 1:S23-S30. doi: 10.1002/oby.22116. PMID: 29464922.
10. De Moraes, A.C.F., Nascimento-Ferreira, M.V., Forjaz, C.L.d. *et al.* Reliability and validity of a sedentary behavior questionnaire for South American pediatric population: SAYCARE study. *BMC Med Res Methodol* 20, 5 (2020). https://doi.org/10.1186/s12874-019-0893-7.
11. Medeiros-Oliveira, Vanessa Cassia ; Viana, Raytta Silva ; Oliveira, Ariclecio Cunha De ; Nascimento-Ferreira, Marcus Vinicius ; De Moraes, Augusto César Ferreira . Are sleep time and quality associated with inflammation in children and adolescents? A systematic review. PREVENTIVE MEDICINE REPORTS JCR, v. 35, p. 102327-1, 202.
12. Nascimento-Ferreira, M. V.; Collese, T. ; Moraes AC e de Moraes AC ; Rendo-Urteaga, T. ; Moreno, L. A. ; Carvalho, H. B. . Validity and reliability of sleep time questionnaires in children and adolescents: A systematic review and meta-analysis. Sleep Medicine Reviews JCR, v. 30, p. 85-96, 2016.
13. Nascimento-Ferreira MV, de Moraes ACF, Monsalve-Álvarez JM, Tello F, Araujo-Moura K, Delgado CA, Bove MI, Moreno LA, Carvalho H. Psychometric properties of 4-item questionnaire for sleep habits and time in a South American paediatric population. Sleep Sci. 2021 Apr-Jun;14(2):169-174. doi: 10.5935/1984-0063.20200048. PMID: 34381581; PMCID: PMC8340887.
14. Rendo-Urteaga T, Saravia L, Sadalla Collese T, Monsalve-Alvarez JM, González-Zapata LI, Tello F, Martínez-Oliván B, Torres-Leal FL, Moreno LA, De Moraes ACF, Carvalho HB. Reliability and validity of an FFQ for South American children and adolescents from the SAYCARE study. Public Health Nutr.

2020 Jan;23(1):13-21. doi: 10.1017/S1368980019002064. Epub 2019 Sep 12. PMID: 31511116; PMCID: PMC10200494.
